# A comparison of visual identification of dental radiographic and nonradiographic images using eye tracking technology

**DOI:** 10.1002/cre2.249

**Published:** 2019-10-18

**Authors:** Michael G. Botelho, Manikandan Ekambaram, Sangeeta Y. Bhuyan, Andy Wai Kan Yeung, Ray Tanaka, Michael M. Bornstein, Kar Yan Li

**Affiliations:** ^1^ Prosthodontics, Faculty of Dentistry The University of Hong Kong Hong Kong SAR China; ^2^ Paediatric Dentistry, Faculty of Dentistry University of Otago Dunedin New Zealand; ^3^ Oral and Maxillofacial Radiology, Applied Oral Sciences, Faculty of Dentistry The University of Hong Kong Hong Kong SAR China; ^4^ Centralized Research Laboratories, Faculty of Dentistry The University of Hong Kong Hong Kong SAR China

**Keywords:** eye tracking, face recognition, gaze analysis, visual recognition

## Abstract

**Objectives:**

Eye tracking has been used in medical radiology to understand observers' gaze patterns during radiological diagnosis. This study examines the visual identification ability of junior hospital dental officers (JHDOs) and dental surgery assistants (DSAs) in radiographic and nonradiographic images using eye tracking technology and examines if there is a correlation.

**Material and methods:**

Nine JHDOs and nine DSAs examined six radiographic images and 16 nonradiographic images using eye tracking. The areas of interest (AOIs) of the radiographic images were rated as easy, medium, and hard, and the nonradiographic images were categorized as pattern recognition, face recognition, and image comparison. The participants were required to identify and locate the AOIs. Data analysis of the two domains, entire slide and AOI, was conducted by evaluating the eye tracking metrics (ETM) and the performance outcomes. ETM consisted of six parameters, and performance outcomes consisted of four parameters.

**Results:**

No significant differences were observed for ETMs for JHDOs and DSAs for both radiographic and nonradiographic images. The JHDOs showed significantly higher percentage in identifying AOIs than DSAs for all the radiographic images (72.7% vs. 36.4%, *p* = .004) and for the easy categorization of radiographic AOIs (85.7% vs. 42.9%, *p* = .012). JHDOs with higher correct identification percentage in face recognition had a shorter dwell time in AOIs.

**Conclusions:**

Although no significant relation was observed between radiographic and nonradiographic images, there were some evidence that visual recognition skills may impact certain attributes of the visual search pattern in radiographic images.

## 1 INTRODUCTION

An early identification of an abnormality suggesting pathological change is a critical skill for every health‐care professional. Such an early identification using visual or special investigations such as radiological examination can aid not only in early diagnosis of a disease but also more importantly in the early initiation of therapy to treat the underlying condition.

Diagnostic errors in the accuracy and interpretation of medical images have been reported since the 1940s, and abnormalities have been either missed or over‐read with various rates across numerous experimental studies (Cooper, Gale, Darker, Toms, & Saada, 2009). The misinterpretation of chest radiograph from missing subtle early changes has led to delay in diagnosis and initiation of treatment (Lee, Nagy, Weaver, & Newman‐Toker, 2013; Turkington, Kennan, & Greenstone, 2002).

In dentistry, among special investigations, radiological diagnosis is one of the key cornerstones for accurate diagnosis and subsequent patient care. The radiographic evaluation of the periapical area by dentists has been reported to be unpredictable and inconsistent regarding the diagnosis of pulpal and bone disease (Sherwood, 2012). Errors in identification of abnormalities account for a major part of misdiagnosis in radiology and can result from cognitive biases or a faulty visual search (Van der Gijp et al., 2017). Variability in radiologists' performances may occur for several reasons, including differences in decision making and recognition abilities. In turn, these abilities may be influenced by variability in training and experience or preexisting individual differences in perceptual abilities (Sunday, Donnelly, & Gauthier, 2017).

In dental education until now, training on radiological diagnosis has been done by several conventional models, such as master‐apprenticeship model, lectures, and case discussions in small groups. However, new technologies allow different methods for understanding the subject and enhancing the teaching and learning methods. Despite the widespread use of eye tracking in other disciplines, there has been surprisingly little use in dental research and education. Tracking of visual search parameters such as dwell time, gaze pattern, and gaze duration has been shown to help in understanding the reasons behind false positive and false negative radiological diagnosis (Brunyé, Drew, Weaver, & Elmore, 2019; Krupinski, Chung, Applegate, DeSimone, & Tridandapani, 2016). Despite extensive research on eye tracking of radiographic images from various medical disciplines, limited work has been done till date on eye tracking of dental radiographic images in identification of teeth and jaw bone‐related pathologies. Eye tracking could be a valuable tool in dental education for training students to prevent false positive and false negative results by tracking their radiographic search pattern. Tracking visual search patterns of students on radiographic images can potentially generate large data sets, which can help in understanding the reasons for errors in misdiagnosis. This in turn can aid in improving teaching and learning methods on how to prevent such errors.

There is a common observation that students have different ability to observe and diagnose normal and abnormal clinical conditions despite similar training and experience. One possible hypothesis is that some students may have a greater inherent ability of pattern recognition for identification. The extent to which individuals in the normal population vary in perceptual ability is largely unknown, but recent studies have shown large individual differences in perceptual processing of faces, of various familiar object categories, and even of novel objects (Sunday et al., 2017). Research in psychology has shown that people differ in their ability in facial recognition or pattern identification, whereas some inherently have superior ability compared with others (Bobak, Bennetts, Parris, Jansari, & Bate, 2016). Russell, Duchaine, and Nakayama (2009) in his study provided evidence for the existence of people with exceptionally good face recognition ability by identifying a group of individuals who outperformed control participants on tests of face memory, face perception, and familiar face recognition. It is not known if such particular ability observed in face recognition may be also repeated for identifying radiographic abnormalities or anomalies.

The aim of the present study was to determine if the ability to visually identify particular targets or abnormalities in nonradiographic images affords greater skills in the identification of the dental radiographic abnormalities or anomalies using the eye tracking technology. We also provide some recommendations/considerations for future eye tracking studies (Sunday et al., 2017).

The following hypotheses were tested in the study:

1 There would be no difference in identification and eye tracking parameters between dentists and assistants while assessing dental radiographic and nonradiographic images.
2 The enhanced ability to identify anomalies or targets on nonradiographic images will translate to the ability to identify abnormalities on dental radiographic images.

## 2 MATERIAL AND METHODS

This study protocol was reviewed and approved by the Institutional Review Board of the University of Hong Kong/Hospital Authority Hong Kong West Cluster (UW 16‐215). An informed written consent was obtained from each participant in the study. This pilot investigation was undertaken with two readily available cohorts of dental personnel, namely, dentists and dental surgery assistants (DSAs).

### 2.1 Subjects

Junior residents, with the working title of junior hospital dental officers (JHDOs), and DSAs from the Faculty of Dentistry were invited to be participants in the present study on a voluntary basis. The JHDOs were in their first year of work and the DSAs had a minimum of 15 years of experience. These two groups were selected as convenient samples, and subsequently, nine DSAs and nine JHDOs were recruited.

### 2.2 Images

Twenty digital panoramic radiographs were selected and anonymized from the patient database of Oral and Maxillofacial Radiology, Faculty of Dentistry, HKU. Inclusion criteria were good image quality as perceived by the expert panel and with one to three abnormalities. Exclusion criteria included presence of distractors that may affect the natural eye tracking pattern of participants. Therefore, radiographs with multiple missing teeth, amalgam fillings, crowns and bridges, and presence of obvious radiographic errors were not included. A panel of five experts consisting of specialists from Oral and Maxillofacial Radiology, Pediatric Dentistry and Prosthodontics selected a total of six panoramic images from the initial sample of 20. Five of these contained a total of 11 anomalies/abnormalities, known as areas of interest (AOIs; Table 1), whereas one panoramic image was normal. Each AOI was categorized by the panel of experts over three meetings as easy, medium, and hard. All the images were shown using a software with no manipulation of the contrast, brightness, and magnification. Therefore, all the images were preprocessed for contrast and brightness.

**Table 1:** Identification and classification of areas of interests (AOIs) in five panoramic images

Category	Type of AOI	Number of AOIs	Kappa value
Easy	Ameloblastoma	7	.665
	Supernumerary tooth		
	Impacted permanent tooth		
	Impacted wisdom tooth (×2)		
	Dentigerous cyst (×2)		
Medium	Periapical inflammatory lesion	3	
	Radicular cyst		
	Supernumerary tooth		
Hard	Supernumerary tooth	1	

To identify potential super pattern recognizers who are good at visual identification, we tested the subjects with 16 nonradiographic images, among which 11 were dealing with pattern recognition, three with face recognition, and the remaining two with image comparison. Ultimately, this study consisted of six radiographic and 16 nonradiographic images. The radiographic and nonradiographic images were shown in the same random order to each participant.

### 2.3 Eye tracking procedure

The RED‐m (Sensomotoric Instruments, Teltow, Germany) system was used to track the eye movements. The operating distance between the device and an observer's eyes was between 50 and 75 cm. The system has a gaze position accuracy of 0.5° and a spatial resolution of 0.1°. A 9‐point initial on‐screen calibration was used for each participant that was followed by a 4‐point calibration to confirm the preliminary calibration. The process was repeated until an accuracy value of 0.5° was obtained.

During the experiment, each participant was invited to sit inside a quiet seminar room with normal illumination. The aim of the experiment was explained to all participants, and a written consent form was obtained from each participant. The subject would face the wall with a laptop computer screen placed in front of him or her on the table. The operating distance between the device and the observer's eyes was between 50 and 75 cm. Instructions were displayed on the screen, indicating that there was no time limit for the experiment and the images could contain none, single, or multiple AOIs. Whenever a participant identified an AOI, he or she had to gaze at the AOI and left click on the mouse. The system then recorded the answer. Subjects were explicitly explained that their task was to identify the presence and location of the AOIs without the need to recognize the identity of the AOIs.

**Figure 1:**
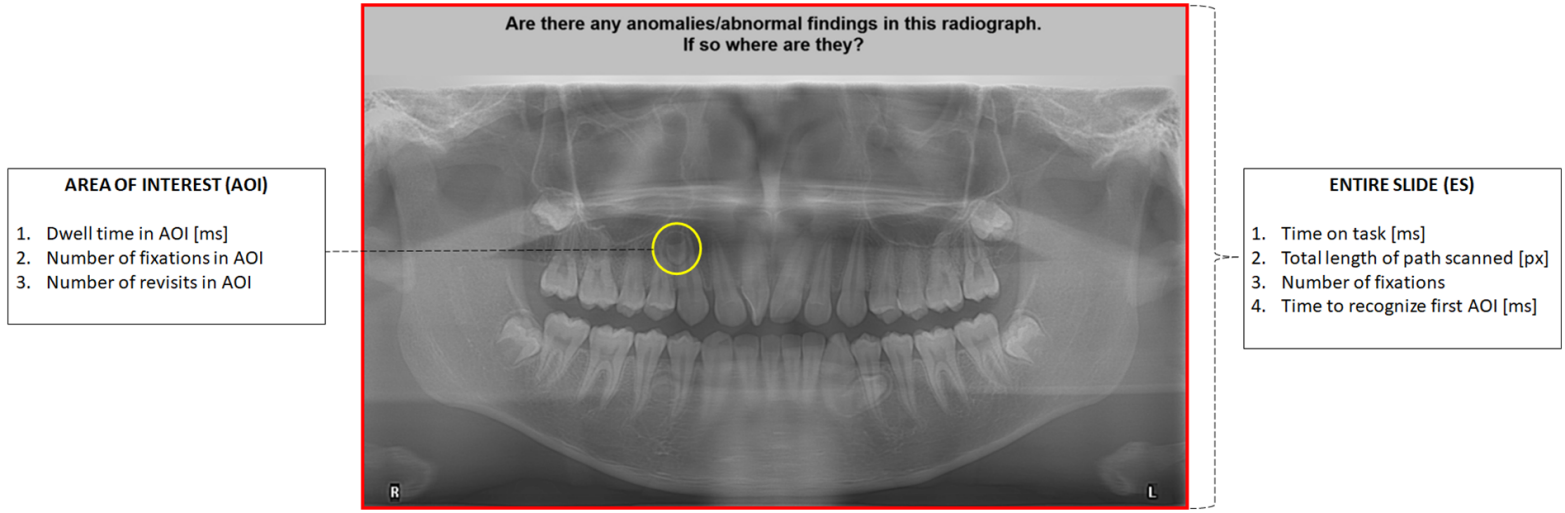
Eye tracking metrics defining the two main measurement domains of area of interest (AOI) and entire slide (ES). There are six different parameters used to measure the AOI or ES (Table 2), with one common variable to both—number of fixations

For data analysis, two main measurement domains were selected—the entire slide and AOI (Figure 1). The AOI is defined as the area of the abnormality on the panoramic images, whereas the entire slide refers to the whole panoramic radiographic/nonradiographic image and the question template. To evaluate these two domains, two analytics were defined—the eye tracking metrics (ETM) and the performance outcomes (Table 2). The ETM parameters are described and defined in Table 3. The performance outcomes were measured as the correct identification percentage and number of incorrect identifications of the radiographic and nonradiographic images. Incorrect identifications were made up of number of false positive answers and number of missed responses. The data set of the ETM and the performance outcomes for each participant was exported from BeGaze (Sensomotoric Instruments) into Excel (Microsoft Corp., Redmond, WA, USA), where they were grouped according to participant type (DSAs and JHDOs). These data were imported into IBM SPSS Version 24 for further statistical analyses.

**Table 2:** The domains and parameters selected to be investigated

Domain	Eye tracking metrics	Performance outcome
Entire slide	1. Total time spent on task (s) 2. Total length of path scanned (px) 3. Number of fixations 4. Time to recognize the first AOI (s)	1. Correct identification percentage (for nonradiographic images only) 2. Number of incorrect identification (is made up of 3 and 4) 3. Number of false positive response 4. Number of missed response
Area of interest (AOI)	1. Dwell time in AOI (s) 2. Number of fixations in AOI 3. Number of revisits in AOI	1. Correct identification percentage in AOI 2. Number of incorrect identification (is made up of 3 and 4) 3. Number of false positive response 4. Number of missed response

**Table 3:** Definitions of eye tracking metrics parameters measured for the two domains—area of interest (AOI) and entire slide (ES)

S. no.	Parameter	Domain	Definition	Diagrammatic representation
1.	Time on task (ms)	ES	Quantifies the amount of time that participants spent looking at the entire slide	(A = start point, B = end point) 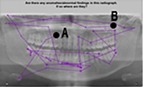 Total time spent from start point A to end point B
2.	Total length of path scanned (px)	ES	Total distance of the participants' eye scanpath over the entire slide	(A = start point, B = end point) 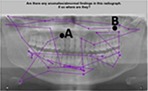 Total distance from start point A to end point B
3.	Time to recognize first AOI (ms)	ES	The time taken to first identify an AOI	(A = start point, 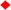 = participant identification of the AOI) 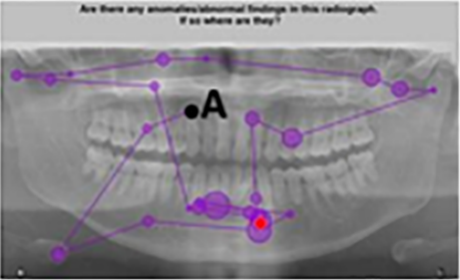 Time to reach the red diamond from start point A
4.	Number of fixations	ES and AOI	Number of points at which the eye scanpath pauses (≥80 ms), which is represented by the circles along the eye scanpath	(A = start point, B = end point, 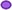 = fixations) 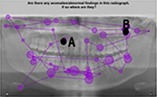 Total number of all the purple circles
5.	Dwell time (ms)	AOI	Dwell time is the amount of time spent looking within an AOI (s)	( 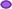 = fixations, 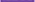 = saccades) 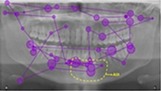 Sum of durations of all fixations and saccades that hit the AOI
6.	Number of revisits in AOI	AOI	It is the number of times the participants' eye tracking revisits an AOI after first fixation on the AOI	

### 2.4 Statistical analysis

Fleiss' kappa was calculated to evaluate the inter‐observer agreement on the difficulty level of the AOIs in the radiographs among the five experts. The final decision of difficulty level was set as the mode (the largest agreement) among the five experts. Shapiro–Wilk test was performed as normality test. If the data did not follow normal distribution, then the data would be presented in median value in JHDOs and DSAs. Nonparametric Mann–Whitney test was performed for each parameter to compare between JHDOs and DSAs in radiographic and nonradiographic images separately. If the overall comparison was significant in AOI of radiographic image, further comparison in AOI of radiographic images with three difficulty levels (easy, medium, or hard) would also be performed. If the overall comparison was significant in nonradiographic image, further comparison in the three types of nonradiographic images (pattern recognition, face recognition, and image comparison) would also be performed.

Spearman's correlation was used to investigate the relationship between correct identification percentage in the three types of nonradiographic images (pattern recognition, face recognition, and image comparison) and the radiographic parameters in JHDOs and DSAs, respectively. For further correlation, the AOI parameters were classified based on their difficulty levels as easy, medium, and hard. Given the number of comparisons in the number of parameters and subgroups, we used the Bonferroni correction and set the significance level as .05. All of the tests were performed with two‐tailed test by IBM SPSS Statistics for Windows Version 24 (IBM Corp., Armonk, NY, USA).

## 3 RESULTS

### 3.1 Types of AOI

The six panoramic images used in this study contained a total of 11 AOIs. Table 1 depicts the categorization of the AOIs (by difficulty level). Fleiss' kappa value for AOI categorization was .665.

### 3.2 Descriptive data of participants

The study participants were nine JHDOs and nine DSAs. The JHDOs were four males and five females in the age group of 25–26 years with less than 1 year of work experience. The DSAs were all females aged between 36 and 55 years (mean age = 39.55) with a work experience of 15 to 30 years.

### 3.3 Comparisons on the descriptive data of the parameters in JHDOs and DSAs

The ETMs (as defined in Table 3) showed no significant difference (*p* > .05) for JHDOs and DSAs (Tables 4 and 5) for both radiographic and nonradiographic images. One notable finding in the nonradiographic images was that the JHDOs had a high correct identification percentage with more time spent on task and a longer path scanned compared with the DSAs; however, this difference was not statistically significant. For the radiographs, JHDOs had significantly higher percentage in identifying AOIs than DSAs for all the radiographs (72.7% vs. 36.4%, *p* = .004) and for the easy categorization of radiographic AOIs (85.7% vs. 42.9%, *p* = .012; Table 6). Interestingly, for medium and hard radiographs, there was no difference between the two groups. In addition, the JHDOs experienced a longer dwell time and more numbers of fixations in AOIs, although this was not significant (*p* > .05).

**Table 4:** Descriptive data for eye tracking metrics of the entire slide parameters of the six radiographic images and participants (JHDO/DSA)

Parameter	JHDO Median	DSA Median	*p* value
Total time on task (s)			
Overall radiograph	30.0	18.6	1.000
Normal	33.5	36.7	
Total length of path scanned (px)			
Overall radiograph	10,034.0	9,507.5	1.000
Normal	12,280.0	16,230.0	
Number of fixations			
Overall radiograph	45.8	35.3	1.000
Normal	51.0	63.0	
Time to recognize the first AOI (s)			
Overall radiograph	5.0	6.8	1.000
Normal	NA	NA	

Abbreviations: AOI, area of interest; DSA, dental surgery assistant; JHDO, junior hospital dental officer; NA, not applicable.

**Table 5:** Descriptive data for eye tracking metrics of entire slide parameters in the 16 nonradiographic images—types of nonradiographic images (pattern recognition, face recognition, and image comparison) and participants (JHDO/DSA)

Parameter	JHDO Median	DSA Median	*p* value
Total time on task (s)			
Overall nonradiograph	34.4	27.9	.564
Pattern recognition	39.6	32.0	
Face recognition	30.9	18.1	
Image comparison	24.6	24.6	
Total length of path scanned (px)			
Overall nonradiograph	15,876.4	12,696.5	1.000
Pattern recognition	16,675.2	12,878.6	
Face recognition	12,511.7	10,841.0	
Image comparison	24,310.5	17,413.0	
Number of fixations			
Overall nonradiograph	63.9	52.0	.966
Pattern recognition	64.4	58.9	
Face recognition	52.3	39.3	
Image comparison	65.5	48.0	
Time to recognize the first AOI (s)			
Overall nonradiograph	19.6	10.9	.966
Pattern recognition	22.6	13.5	
Face recognition	16.1	15.3	
Image comparison	0.0	0.0	

Abbreviations: AOI, area of interest; DSA, dental surgery assistant; JHDO, junior hospital dental officer.

**Table 6:** Descriptive data of the parameters for the area of interest (AOI) in radiographic images—degree of difficulty (easy, medium, and hard) and participants (JHDO/DSA)

Parameter	JHDO Median	DSA Median	*p* value
Dwell time in AOI (s)			
Overall	0.64	0.61	1.000
Easy	0.83	0.73	
Medium	0.44	0.30	
Hard	0.33	0.00	
Number of fixations in AOI			
Overall	1.55	1.09	.644
Easy	2.14	1.43	
Medium	1.00	0.33	
Hard	1.00	0.00	
Number of revisits in AOI			
Overall	0.36	0.36	1.000
Easy	0.43	0.29	
Medium	0.33	0.00	
Hard	0.00	0.00	
Correct identification percentage in AOI			
Overall	72.7%	36.4%	.004*
Easy	85.7%	42.9%	.012*
Medium	66.7%	0.0%	.924
Hard	100.0%	0.0%	1.000

Abbreviations: DSA, dental surgery assistant; JHDO, junior hospital dental officer.

* Significant at *p* < .05.

### 3.4 Association between nonradiographic and radiographic eye tracking performances

Spearman's correlation tests have shown that the correct identification percentages in the three types of nonradiographic images (pattern recognition, face recognition, and image comparison) had no significant correlation with the radiographic parameters in JHDOs and DSAs apart from JHDOs having a higher correct identification percentage in face recognition would have a shorter dwell time in AOI in overall (rho = −.80, *p* = .039; Tables 7 and 8). Negative Spearman correlation (−.13, *p* > .05) suggests that the JHDOs who were better in face recognition had shorter time to identify the first AOI. This implies that the JHDOs who were better in face recognition identified the first AOI quickly, though it was not statistically significant. Table 9 shows the performance outcomes for the nonradiographic images.

**Table 7:** Spearman's correlation showing the relationship between correct identification percentage in the three categories of the nonradiographic images (pattern recognition, face recognition, and image comparison) and the parameters selected (only significant parameters shown) in radiographic images in JHDO and DSA

Parameter		JHDO			DSA		
Dwell time in AOI (s)		Pattern recognition	Face recognition	Image comparison	Pattern recognition	Face recognition	Image comparison
Overall	Correlation	−.03	−.80*	.20	−.32	.15	−.19
	*p* value	1.000	.039*	1.000	1.000	1.000	1.000
Easy	Correlation	.25	−.60	.22	−.30	.22	−.15
	*p* value	1.000	1.000	1.000	1.000	1.000	1.000
Medium	Correlation	−.28	−.83	.06	−.29	.28	−.41
	*p* value	1.000	.069	1.000	1.000	1.000	1.000
Hard	Correlation	−.10	−.08	.14	−.26	.06	−.33
	*p* value	1.000	1.000	1.000	1.000	1.000	1.000

Abbreviations: AOI, area of interest; DSA, dental surgery assistant; JHDO, junior hospital dental officer.

* Significant at *p* < .05.

**Table 8:** Spearman's rho correlations in performance outcomes between six radiographic image and 16 nonradiographic image parameters

Parameter		JHDO			DSA		
Performance outcome		Pattern recognition	Face recognition	Image comparison	Pattern recognition	Face recognition	Image comparison
Correct identification percentage	Correlation	−.42	−.48	−.19	−.20	−.56	−.34
	*p* value	1.000	.751	1.000	1.000	.455	1.000
Number of incorrect identification	Correlation	.10	−.29	.55	.00	.15	.31
	*p* value	1.000	1.000	.514	1.000	1.000	1.000
Number of false positive answers	Correlation	.42	.39	.26	.20	.56	.42
	*p* value	1.000	1.000	1.000	1.000	.455	1.000
Number of missed responses	Correlation	−.55	−.61	−.10	−.20	−.56	−.34
	*p* value	.485	.333	1.000	1.000	.455	1.000

Abbreviations: DSA, dental surgery assistant; JHDO, junior hospital dental officer.

**Table 9:** Descriptive data for performance outcomes of the 16 nonradiographic images—types of nonradiographic images (pattern recognition, face recognition, and image comparison) and participants (JHDO/DSA)

Performance outcome	Nonradiographic image	JHDO Median	DSA Median	*p* value
Correct identification percentage	Overall nonradiographs	69.8%	57.3%	1.000
	Pattern recognition	65.2%	63.6%	
	Face recognition	66.7%	66.7%	
	Image comparison	50.0%	100.0%	
Number of incorrect identification	Overall nonradiographs	1.3	1.3	1.000
	Pattern recognition	1.5	1.5	
	Face recognition	1.0	1.0	
	Image comparison	1.0	1.0	
Number of false positive answers	Overall nonradiographs	0.4	0.6	1.000
	Pattern recognition	0.4	0.6	
	Face recognition	0.3	0.3	
	Image comparison	0.5	0.0	
Number of missed responses	Overall nonradiographs	0.9	0.8	1.000
	Pattern recognition	1.1	0.8	
	Face recognition	0.7	0.7	
	Image comparison	0.5	1.0	

Abbreviations: DSA, dental surgery assistant; JHDO, junior hospital dental officer.

### 3.5 Association between normal radiograph and the radiographs with abnormalities

For both the groups, the number of false positive responses in normal radiograph was higher than the radiographs with abnormalities, although there was no statistically significant difference between the groups (Table 10). Interestingly, both groups had more number of fixations, a longer path scanned, and more time spent on the normal radiograph than the radiographs with anomalies/abnormalities.

**Table 10:** Descriptive data for performance outcomes of the six radiographic images (with abnormalities and normal) and participants (JHDO/DSA)

Performance outcome	Radiographic image	JHDO Median	DSA Median	*p* value
Correct identification percentage	Overall radiograph	42.4%	26.2%	.160
	Radiographs with abnormalities	42.4%	26.2%	.160
Number of incorrect identification	Overall radiograph	3.8	4.0	.160
	Radiograph with abnormalities	4.6	4.6	1.000
	Normal radiograph	0.0	1.0	.160
Number of false positive answers	Overall radiograph	2.3	3.2	.124
	Radiograph with abnormalities	2.8	3.6	.252
	Normal radiograph	0.0	1.0	.160
Number of missed responses	Overall radiograph	1.5	0.8	.252
	Radiograph with abnormalities	1.8	1.0	.252

Abbreviations: DSA, dental surgery assistant; JHDO, junior hospital dental officer.

## 4 DISCUSSION

Pattern recognition is a process in which we use multiple senses in order to make decisions. Visual experts believe that in order to improve performance, one must build a mental repertoire of patterns of normality and abnormality (Hanley et al., 2017). Super recognizers have an above average ability to recognize faces, typically the top 1–2% on a face recognition tests. Gauthier, Tarr, Anderson, Skudlarski, and Gore (1999) and Kanwisher, McDermott, and Chun (1997) investigated specific areas of the brain in relation to face recognition abilities. These higher cortical areas are located on the inferior surface of the temporal lobe and have a significantly larger surface area, compared with other cortical regions (Gauthier et al., 1999; Kanwisher et al., 1997). Al‐Imam, Ali, and Saad (2018) evaluated the face recognition abilities of the medical students and reported 51.5% of students to be potential super recognizers with significant difference between males and females.

The main aim of this pilot study was to test the hypothesis that individuals have different capability in identifying and diagnosing radiographic anomalies/abnormalities on dental radiographs and that this may be related to an inherent pattern recognition that individuals may have. However, the overarching hypothesis of this pilot study was not supported, although there was a statistically significant correlation between the correct identification percentage in face recognition group of the nonradiographic images and the dwell time in the AOIs (overall) in the radiographic images. The JHDOs with higher correct identification percentages in face recognition had shorter dwell time in the AOI of the radiographic images. The relation of these is difficult to interpret but may suggest that there are some inherent visual recognition skills that can impact on other visual pattern searching attributes. Further analysis of face recognition to image searching patterns should be performed again with a larger sample size to explore this relation. A number of factors were identified during the capture and analysis of data from which we propose recommendations or guidelines so that others may conduct more robust eye tracking studies (Table 11).

**Table 11:** Recommendations and guidelines for a more robust eye tracking study

Considerations/recommendations for an effective eye tracking study	
Data capture	• Participants should be provided with written and then verbal instructions prior to the study to ensure understanding. • Participants should be able to ask questions after the explanation. • One example question should be displayed on the screen prior to starting the test to familiarize participants with the test requirements. • Participants should be watched during the testing to ensure they gaze and click simultaneously at the AOI to register their response. • Field notes should be taken during the testing conditions to record anomalous performances. • Question stem and the radiograph/image should be on separate slides. • Simplify the question setup—questions should be specific and self‐explanatory. • Choose appropriate eye tracking metrics that suit the research objectives, as there are many to choose from.
Ambient factors	• Have a clean computer screen and adjust screen brightness/contrast for the images and ambient lighting prior to the study • Use of chin rest to limit head movements and accuracy of eye tracking • Have computer screen facing a blank wall to avoid distractions • Keep waiting participants away from test environment to avoid distraction

Abbreviation: AOI, area of interest.

Suwa, Furukawa, Matsumoto, and Yosue (2001) reported a longer time spent and more number of fixations in the normal images on analyzing the eye movement of dentists during their reading of the CT images of head and neck region. This is supported by Turgeon who examined panoramic images reported with dental students and oral and maxillofacial radiologists who spent longer search times, covered greater distances, and had greater number of eye fixations for normal images than images of pathoses (Turgeon & Lam, 2016). This is similar to our study with participants spending more time in normal images with more number of fixations and a longer path scanned. This finding may reflect that more time is spent looking at normal radiographs to identify an AOI or because of the testing environment; participants spend longer searching for something to identify.

In the current study, the JHDOs were better at identification percentages in the overall and easy radiographs; this may be expected given the training afforded albeit with a shorter period of practice their recent graduation. However, the DSAs with 15 years of experience may have had acquired a particular skill set in pattern recognition in identifying radiographic abnormalities that they have acquired vicariously observing radiographic diagnosis in a teaching clinic. However, it is to be remembered that the diagnosis of the lesions was not part of the current study and therefore, a greater degree of difference would have been expected in the diagnosis differences.

There are many parameters that can be captured and analyzed using the eye tracking technology. These parameters are not universal; many of them are useful only for specific purposes. Depending on the task, one needs to choose proper ETM to reveal features related to the aims of the analysis. The most common eye tracking parameters are based on fixations and/or saccades. A fixation is the amount of time that the participant's gaze remains still, and the number of fixations may reflect a more careful scrutiny of the image and attention to a particular area in the image. A saccade is defined as a small rapid change in gaze location from one fixation to another so that a fixation is regarded as being bordered by two saccades. However, in the current study, we did not analyze saccades as this is a kind of proxy for fixation as a saccade is bounded by two fixations. There are also ETM applying both saccades and fixations called scanpath. The scanpaths analysis provides insights into how individuals prioritize locations of semantic interest although the analysis of these paths is difficult. A longer scanpath may correspond to a more detailed image searching and a more methodical search pattern. In addition, time metrics can be added to these to determine the amount of time taken with particular metrics.

The present study also evaluates the time on task with no significant differences between the two groups. The time on task correlates to an attentive and a systematic methodological approach in searching the image. It has been observed that in general, the visual search time decreases with increasing levels of expertise (Giovinco et al., 2015; Rubin et al., 2014; Wood et al., 2013), although in some studies time on task did not significantly differ between different experience levels (Donovan & Litchfield, 2013; Mallett et al., 2014).

In this present study, the Fleiss' kappa value of .665 among the five experts implied substantial inter‐observer agreement on the difficulty level of the AOIs in the radiographs (Cohen, 1960). Similarly, a previous study has evaluated the inter‐observer agreement of assessing the developmental stage of third molars on panoramic radiographs and reported kappa values of .52–.68, which can be considered as comparable with the kappa value of the present study (Dhanjal, Bhardwaj, & Liversidge, 2006).

It is difficult to source radiographs with only one AOI, and as such, there were between one and three AOIs on the radiographs that may be considered more authentic to the clinical cases. There are limitations in the present study and therefore termed a pilot study. The major limitation is a small cohort of participants in the study. Furthermore, these observers varied greatly in radiographic interpretation expertise. Also, the present study reported where participants' eyes gazed within the radiographs but did not demonstrate their cognitive interpretations. Observers' interpretations may not necessarily be associated with their gaze fixations (Drew & Williams, 2017). Eye tracking research alone cannot fully explain radiographic interpretation; both perceptual and cognitive processes are necessary and can be considered with doing a talk aloud review after performing the eye tracking.

## 5 CONCLUSION

The present pilot eye tracking study has presented the ability to identify AOIs in radiographic and nonradiographic images. Although no significant relation was observed, there was some evidence that face recognition may impact certain attributes of eye tracking on radiographic images. Further studies are needed to explore this phenomenon.

## CONFLICT OF INTEREST

The authors declare no conflict of interest.
